# Functional MRI evidence of brain alterations in premenstrual dysphoric disorder: a systematic review

**DOI:** 10.3389/fpsyt.2026.1795420

**Published:** 2026-06-04

**Authors:** Kornelia Bartoszewicz, Edyta Szurowska

**Affiliations:** 1 Student Scientific Circle Interdisciplinary Health Care Management at the Department of Public Health & Social Medicine and 2nd Division of Radiology, Faculty of Health Sciences with the Institute of Maritime and Tropical Medicine, Medical University of Gdańsk, Gdańsk, Poland; 2 2nd Department of Radiology, Medical University of Gdansk, Gdańsk, Poland; 3 Department of Radiology, University Clinical Centre, Gdańsk, Poland

**Keywords:** fMRI, large-scale brain networks, menstrual health, neuroimaging, PMDD, women’s health

## Abstract

**Introduction:**

Premenstrual Dysphoric Disorder (PMDD) affects approximately 1.6% of women of reproductive age and significantly impacts quality of life. Despite its prevalence, the underlying pathophysiology remains incompletely understood. First-line treatment typically involves selective serotonin reuptake inhibitors (SSRIs); however, approximately 40 percent of women with PMDD do not respond to these medications. This systematic review synthesizes current evidence on functional brain alterations in women with PMDD, as assessed using functional magnetic resonance imaging (fMRI), with the goal of identifying potential novel therapeutic strategies.

**Methods:**

Data from 598 participants, including 294 PMDD patients and 304 healthy controls, were analyzed.

**Results:**

The findings suggest alterations in both topdown regulatory mechanisms and large-scale brain networks, including the salience network, default mode network, and executive control network. These alterations are characterized by decreased activation in the anterior cingulate cortex, dorsolateral prefrontal cortex, medial orbitofrontal cortex, and postcentral gyrus, alongside increased activation in the amygdala and insula, as well as impairments in corticolimbic connectivity.

**Discussion:**

These results highlight the complexity of PMDD, implicating widespread neural circuits rather than a single localized dysfunction. Targeting these mechanisms may inform the development of novel interventions for symptom relief.

**Systematic review registration:**

https://www.crd.york.ac.uk/PROSPERO/, identifier CRD420251174749.

## 1 Introduction

It is estimated that 1.6% of women of reproductive age meet the criteria for Premenstrual Dysphoric Disorder (PMDD) (1). Premenstrual dysphoric disorder sits within a broader landscape of menstrual health concerns affecting women globally. Premenstrual symptoms of varying severity affect an estimated 47.8% of reproductive-aged women worldwide, with PMDD representing the most severe end of this spectrum (2). Recognition of menstrual health as a distinct domain of women’s health, encompassing physical, mental, and social well-being in relation to the menstrual cycle, has only recently emerged as a research and policy priority, and disorders such as PMDD remain underdiagnosed and undertreated in many healthcare contexts (3, 4). Both ICD-11 and DSM-5 define PMDD by a pattern of affective, somatic, and cognitive symptoms that begin several days before the onset of menses, start to improve within a few days after onset of menses, and become minimal or absent within approximately one week following menses (5, 6). Symptoms must be present in most menstrual cycles over the preceding year and must cause significant distress or impairment in personal, family, social, educational, occupational, or other important areas of functioning. DSM-5 requires confirmation through prospective daily ratings over at least two symptomatic cycles, while ICD-11 recommends prospective documentation. Symptoms cannot be explained by exacerbation of another disorder, such as major depressive disorder, panic disorder, persistent depressive disorder, or a personality disorder, though PMDD may co-occur with any of these.

During the premenstrual period, the impairment due to PMDD may reach the average level of severity of major dysphoric disorders and is associated with a higher suicidal risk (7). Premenstrual Syndrome (PMS) must be differentiated from PMDD. PMS is characterized predominantly by somatic symptoms (e.g., bloating, breast tenderness, headache) that interfere with quality of life, whereas PMDD is defined by severe affective and cognitive symptoms (e.g., marked irritability, depressed mood, anxiety, mood lability) that cause significant distress or functional impairment. This distinction has clinical importance, as PMDD’s affective severity is associated with substantially elevated suicidal risk compared with PMS (7). Due to this fact, the disorder not only lowers the quality of life of affected women but also poses direct danger to their lives. Nonetheless, despite the substantial impact on affected women’s lives, important gaps remain in our understanding of the pathophysiology of this disorder (8).

Current treatment options for PMDD include antidepressants, hormonal therapies, and psychotherapy, with selective serotonin reuptake inhibitors (SSRIs) considered the first-line treatment (9). However, approximately 40% of women with PMDD do not respond to SSRIs, and evidence supporting the effectiveness of alternative options remains limited (10). Identifying abnormalities in brain function could provide targets for the development of future pharmacological or therapeutic interventions.

PMDD is fundamentally a neuropsychiatric disorder and its cyclical emergence is driven by the brain’s response to ovarian steroid fluctuations rather than by abnormal peripheral hormone levels themselves (11). Functional magnetic resonance (fMRI) is particularly well-suited to studying PMDD for several reasons. The capacity of fMRI to capture dynamic, task-evoked neural activity allows direct comparison of brain function across symptomatic and asymptomatic phases within the same individual. Second, PMDD’s symptom profile implicates large-scale networks involved in emotion regulation, salience detection, and cognitive control rather than discrete focal lesions, and fMRI is the modality of choice for characterizing distributed network function. Additionally, fMRI is non-invasive and free of ionizing radiation, making it suitable for repeated measurements across the menstrual cycle in healthy women of reproductive age. Functional MRI (fMRI) often uses the blood-oxygen-level-dependent (BOLD) signal as an indirect measure of neural activity (12). Unlike oxygenated hemoglobin, deoxygenated hemoglobin is paramagnetic, which affects the MR signal. This property forms the basis of BOLD fMRI and is used to estimate patterns of brain activity over time. Thus, the BOLD signal reflects changes in the relative concentration of oxygenated and deoxygenated hemoglobin in the blood that occur following neuronal activation and associated increases in cerebral blood flow. Consequently, BOLD imaging enables researchers to identify brain regions that are more or less active during specific cognitive, emotional, or behavioral tasks.

Therefore, this review aims to summarize evidence on abnormal activity patterns and functional connectivity in women with PMDD, as observed through functional magnetic resonance imaging. It also seeks to relate these neural alterations to the functions of the affected brain regions in order to identify potential mechanisms underlying the symptoms experienced by PMDD patients.

## 2 Methods

This systematic review was prospectively registered with PROSPERO CRD420251174749 and conducted following the Preferred Reporting Items for Systematic Reviews and Meta-Analyses (PRISMA) guidelines and applying the PICO framework, which defined the population as women with PMDD, the intervention as functional magnetic resonance imaging, the control group as healthy individuals, and the outcome as changes in functional neuroimaging measured with functional magnetic resonance. The Covidence software platform was employed to assist in the removing duplicates, screening and data extraction processes. The completed PRISMA 2020 checklist is provided in the **Supplementary Table 1** available in the **Supplementary Materials**.

### 2.1 Search strategy

Relevant articles were identified through the online databases PubMed, EMBASE, and Scopus, with the final search conducted in August 2025. The search strategy used the terms (“PMDD” OR “premenstrual dysphoric disorder”) AND (“functional magnetic resonance” OR “fMRI”). Additionally, the reference lists of included studies were screened to identify eligible articles.

### 2.2 Study selection criteria

The inclusion criteria were: (a) neuroimaging changes in PMDD as the primary outcome; (b) study designs including cross-sectional, prospective, retrospective, case-control, clinical trials; (c) published in English; (d) participants diagnosed with PMDD according to DSM-IV, DSM-IV-TR, or DSM-5 criteria, or prospective daily ratings; and (e) use of functional magnetic resonance imaging. Studies were excluded if they: (a) were literature reviews, editorials, case series, or conference abstracts; (b) involved animal models; (c) included only healthy participants; (d) examined PMDD comorbid with another psychiatric disorder in all subjects; or (e) assessed the effects of therapeutic or hormonal interventions.

### 2.3 Risk of bias assessment

Risk of bias in included studies was assessed using the Newcastle-Ottawa Scale (NOS) for case-control studies (13). For the comparability domain, we pre-specified absence of current hormonal contraceptive use and absence of current Axis-I psychiatric diagnoses as the matching factors. The “exposure” domain was operationalized to refer to PMDD diagnosis, with prospective daily symptom ratings considered the gold-standard ascertainment method. Two reviewers assessed each study independently, with disagreements resolved by discussion. Studies scoring 7–9 stars were classified as low risk of bias, 4–6 as moderate, and 0–3 as high. The full assessment is presented in **Supplementary Table 2**, available in the **Supplementary Materials**.

### 2.4 Data extraction

For each study, the following information was extracted, when available: author, year of publication, sample size, presence of a control group, number of participants in each group, mean age, diagnostic criteria for PMDD, menstrual cycle phases during which neuroimaging was performed, methods used to confirm cycle stage, brain image analysis techniques, regions investigated, evidence of neuroimaging alterations, and task performance, if applicable. Two reviewers independently screened articles and extracted data. Disagreements were resolved through discussion or third-party arbitration.

## 3 Results

### 3.1 Literature search

**Figure 1:**
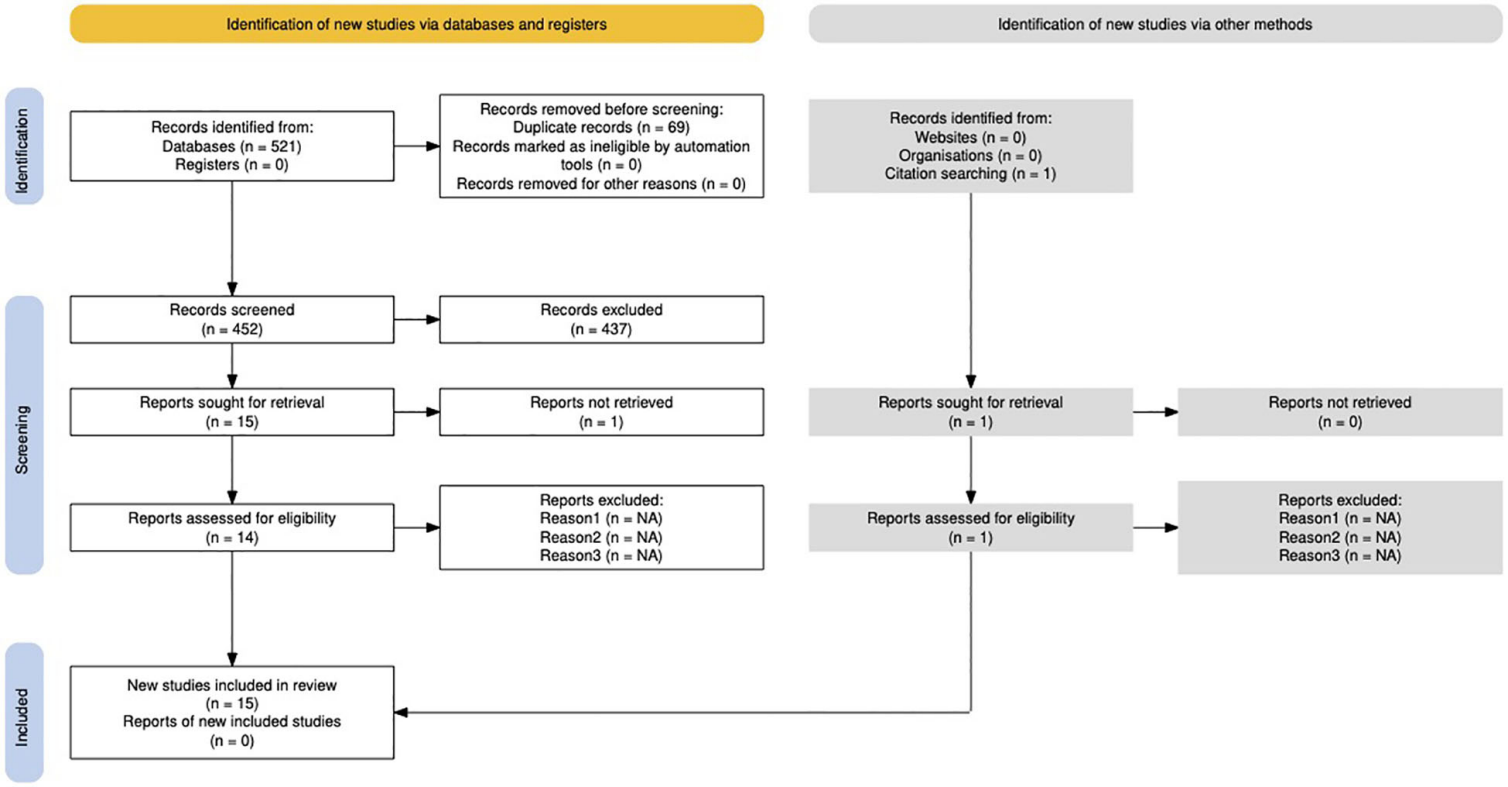
PRISMA 2020 flow diagram of the study identification, screening, and selection process. Flow diagram created using the PRISMA2020 R package (14).

Our initial search identified 521 articles in three databases (PubMed: 22, EMBASE: 62, and Scopus: 437). Additionally, one report identified from a manual search of the reference list was included. In total, 69 duplicate records were removed and 437 articles were excluded as irrelevant after screening titles and abstracts, leaving 16 potentially relevant articles. Of these, one could not be retrieved. Finally, 15 articles were included in this review. A PRISMA diagram of the article selection process is illustrated in **Figure 1**.

### 3.2 Characteristics of the included studies

In this review, a total of 598 participants, including 294 with PMDD and 304 healthy controls (HC), were included. It should be noted that the number of unique participants is lower due to overlapping samples in some of the studies. In this study we included both task-based fMRI (n = 13) and resting-state fMRI imaging (rs-fMRI) (n = 2), with one study using both. An overview of the main characteristics and findings can be found in **Table 1**. Overall methodological quality of included studies as assessed with NOS was good, with 13 of 15 studies scoring 7–9 stars. All studies met the criteria for adequate case definition and comparability of cases and controls on matching factors. The most consistent methodological weaknesses were inadequate reporting of non-response rates and limited descriptions of control selection procedures.

**Table 1:** Main characteristics and findings of the included studies.

Lead author and year	Sample size n (PMDD+, HC)	Neuroimaging technique	Task	Results (PMDD+ compared to HC)	Results (luteal vs. follicular phase, PMDD+)
Baller et al., 2013 (15)	14, 14	task-fMRI	n-back working memory	↑BOLD in the dorsolateral prefrontal cortex bilaterally, the medial frontal gyrus and the cerebellum ↓BOLD in small clusters in the cuneus, precuneus and lateral temporal cortex	NA
Bannbers et al., 2012 (16)	14, 13	task-fMRI	Go/NoGo	↑BOLD in insula in luteal phase ↓BOLD during response inhibition in bilateral inferior parietal lobule, left precuneus, left supramarginal gyrus, right postcentral gyrus, right superior parietal lobule, right caudate body ↓BOLD in left insula in follicular phase	↑BOLD in the insula
Comasco et al., 2014 (17)	16, 15	task-fMRI	emotion processing	↓BOLD in the pregenual anterior cingulate cortex, ventromedial prefrontal cortex independently of menstrual phase	↓BOLD in motor cortex and middle frontal gyrus
Dan et al., 2020 (18)	24, 27	Rs-fMRI	resting-state	↑FC between striatum with temporal cortex and motor cortex, thalamus with amygdala and temporal cortex, pallidum with the temporal cortex ↓FC between temporal cortex with occipital, temporal, motor, and prefrontal regions	NA
Gao et al., 2021 (19)	42, 44	task-fMRI	anger induction, depression induction	↑ReHo in the middle frontal gyrus, temporal lobe, and left cerebellum after anger induction in luteal phase ↑ReHo in the middle frontal gyrus, the middle gyrus and in the cingulate gyrus ↓ReHo in the precuneus, superior frontal gyrus, lobulus paracentralis, and in the right cerebellum after anger induction in luteal phase	NA
Gingnell et al., 2012 (20)	14, 15	task-fMRI	emotion processing	↑BOLD in the amygdala bilaterally in the follicular phase ↑BOLD in the right amygdala in women with PMDD and high trait anxiety in the luteal phase than controls ↑BOLD in the right amygdala in women with PMDD and low trait anxiety in the follicular phase than controls ↓BOLD in the right amygdala in women with high trait anxiety in the follicular phase and ↑BOLD in the luteal phase than women with low scores on trait anxiety	↑BOLD in right amygdala in high anxiety women
Gingnell et al., 2013 (21)	14, 14	task-fMRI	anticipation of emotional stimuli	↑BOLD in medial prefrontal cortex bilaterally and right dorsolateral prefrontal cortex in luteal phase during anticipation of negative stimuli	NA
Gingnell et al., 2014 (22)	14, 13	task-fMRI	exposure to emotional images of negative valence in socially relevant/irrelevant context	↑BOLD in the amygdala and insula in response to social stimuli in the luteal phase ↓BOLD in the anterior cingulate cortex in the luteal phase ↑FC between the amygdala and the anterior cingulate cortex, as well as between the insula and the anterior cingulate cortex	↑BOLD in amygdala in response to social compared to non-social stimuli
Lerner et al., 2024 (23)	17, 20	task-fMRI	induction of empathy	↑NS in anterior insula/inferior frontal sulcus bilaterally and anterior cingulate cortex in the luteal phase ↓NS in bilateral middle frontal sulcus, inferior frontal sulcus, precentral sulcus, superior precentral sulcus, medial prefrontal cortex, precuneus, temporal parietal junction, superior temporal sulcus, inferior temporal sulcus, temporal pole in the luteal phase	NA
Petersen et al., 2018 (24)	18, 18	task-fMRI	emotion regulation	↓BOLD in the right dorsolateral prefrontal cortex and right postcentral gyrus in the luteal phase	↓BOLD in the right precentral gyrus
Petersen et al., 2019 (25)	17, 18	Rs-fMRI, task-fMRI	emotion regulation	↑FC the left executive control network with a region in the left middle temporal gyrus before and after the emotion regulation task	NA
Protopopescu et al., 2008 (26)	8, 12	task-fMRI	emotional linguistic Go/NoGo	↑BOLD in the right amygdala and lateral orbitofrontal cortex and ↓BOLD in medial orbitofrontal cortex in response to negatively valenced stimuli ↓BOLD in the nucleus accumbens in response to positively valenced stimuli In HC there was a premenstrual enhancement in the activity of medial orbitofrontal cortex, PMDD showed the opposite pattern	NA
Reuveni et al., 2023 (27)	24, 27	task-fMRI	emotional face-matching	↑FC of the right amygdala with the right thalamus, the right insula with the right midbrain ↓FC of the posterior cingulate cortex with right middle temporal gyrus, the right amygdala with the left postcentral gyrus, the left amygdala with the bilateral precentral and postcentral gyrus, the left insula with the left cuneus, and the right PPC with the left inferior temporal gyrus, temporo-occipital part and the right PPC with the right cerebellum crus	↓FC between the right posterior cingulate cortex and the left inferior lateral occipital cortex
Stiernman et al., 2023 (28)	29, 27	task-fMRI	emotional discrimination	↑BOLD in bilateral posterior cingulate cortex, left anterior cingulate cortex, left precuneus, bilateral insula, right medial frontal gyrus, right superior frontal gyrus, left supplementary motor area, left postcentral gyrus, dorsal stratum, thalamus and cerebellum in late luteal phase	↑BOLD in right medial frontal gyrus and right superior frontal gyrus, the opposite pattern seen in controls
Stiernman at al., 2025 (29)	29, 27	task-fMRI	emotion regulation	↑BOLD in bilateral dorsal anterior cingulate cortex and cerebellum activity independently of menstrual cycle phase	↑BOLD in amygdala when passive viewing of emotional stimuli

PMDD+, diagnosed with PMDD; HC, healthy controls; ReHo, Regional Homogeneity; Rs-fMRI, resting state fMRI; FC, functional connectivity; NS, neural synchrony. ↑, increased. ↓, decreased.

### 3.3 Main findings

There were significant differences in activation and functional connectivity patterns between women with PMDD and controls, as well as within-group variations depending on the phase of the menstrual cycle (luteal vs. follicular). The results indicate dysfunction in the dorsolateral prefrontal cortex during tasks assessing both cognitive and emotion-regulation processes. Women with PMDD exhibited disrupted activation of the medial and lateral orbitofrontal cortex across the menstrual cycle. Recruitment of the postcentral gyrus was also dysfunctional, with an attenuated response in the right postcentral gyrus and enhanced activity in the left. Several studies indicated aberrant engagement of the anterior cingulate cortex, with the nature of these alterations depending on the task type. A subset of studies further reported disrupted amygdala function, especially in response to socially relevant stimuli. Notably, activation patterns differed between women with low and high trait anxiety, suggesting that trait anxiety may be an important clinical variable. Increased recruitment of the insula during the luteal phase and elevated cerebellar responses both during the luteal phase and independently of menstrual cycle phase were also observed. Functional connectivity patterns were likewise disrupted, with particularly notable alterations in temporal cortex and amygdala connectivity profiles.

## 4 Discussion

### 4.1 Analysis by region

We first examine evidence for region-specific dysfunction in PMDD, before turning to functional connectivity and network-level findings in subsequent sections. The following subsections summarize observations and proposed mechanistic interpretations for each affected brain region.

#### 4.1.1 Dorsolateral prefrontal cortex

The dorsolateral prefrontal cortex (dlPFC) has been examined in several fMRI studies of PMDD, with findings spanning working memory, emotion regulation, and anticipation paradigms. Functionally, the dlPFC is implicated in impulse inhibition, risk-benefit evaluation, decision-making, and working memory, with the left dlPFC more involved in manipulating information within working memory and the right dlPFC supporting broader reasoning processes (30, 31).

In women with PMDD, increased luteal-phase right dlPFC reactivity was observed during the anticipation of negative emotional stimuli compared with healthy controls (20). By contrast, another study showed hypoactivation of the right dlPFC during an emotion regulation task (23). Beyond emotion-related paradigms, working memory studies have also revealed dlPFC abnormalities in PMDD, with greater bilateral activity during n-back performance compared with healthy controls (15). Notably, the magnitude of this overactivation correlated negatively with global functioning, suggesting that increased dlPFC recruitment may reflect inefficient compensatory effort (15).

Taken together, these findings indicate abnormal dlPFC activity in PMDD, with greater recruitment during effortful tasks (anticipation, working memory) alongside reduced activity during active emotion regulation. This dlPFC dysregulation likely contributes to the cognitive symptoms reported by women with PMDD during the symptomatic luteal phase, including concentration difficulties and ‘mental fog’.

#### 4.1.2 Orbitofrontal cortex

The orbitofrontal cortex (OFC) plays a central role in emotional processing by integrating sensory inputs with reward and punishment values in order to guide adaptive behavior. The medial OFC (mOFC) is involved in monitoring, learning, and representing the reward value of stimuli, particularly those associated with subjective pleasantness and hedonic experience, while the lateral OFC (lOFC) is more engaged in evaluating punishment and non-reward (32, 33). Anatomically, the OFC shares extensive reciprocal connections with multiple amygdala nuclei and has been shown to modulate amygdala activity (34, 35).

In healthy individuals, particularly women, mOFC activation increases during the luteal phase in response to negative emotional stimuli, reflecting enhanced top-down modulation of limbic activity that may indicate a compensatory mechanism to maintain emotional regulation when sensitivity to negative affect is elevated (36).

In women with PMDD, this regulatory mechanism appears disrupted. These participants showed reduced mOFC activation in response to negative stimuli during the luteal phase and failed to engage this region under conditions requiring emotional inhibition (26). Simultaneously, lateral OFC activity was increased. This dysfunction likely reflects impaired integration of reward and punishment information and inadequate modulation of amygdalar output, contributing to heightened emotional reactivity and mood instability characteristic of PMDD.

#### 4.1.3 Postcentral gyrus

Although the postcentral gyrus is traditionally recognized as the primary somatosensory cortex responsible for processing tactile input, growing evidence indicates that it also plays a broader role in emotional and cognitive functions, particularly those involving bodily awareness and interoception (37). For instance, participants with lesions in the right somatosensory cortex had impaired ability to rate the intensity of emotions, as well as to recognize, name, and categorize emotions (38).

One study found hypoactivation of the right postcentral gyrus in women with PMDD compared with controls during an emotion regulation task (24). The right postcentral gyrus also showed reduced activity during response inhibition in a Go/NoGo task (16). By contrast, the left postcentral gyrus showed greater activation in PMDD subjects during an emotional discrimination task. These outcomes may indicate disrupted integration of emotional and bodily signals, potentially contributing to difficulties in affect regulation during the luteal phase.

#### 4.1.4 Anterior cingulate cortex

Increasing evidence highlights the crucial role of the anterior cingulate cortex (ACC) in emotional regulation, particularly through its connections with limbic regions such as the amygdala. The ventral ACC is especially involved in top-down inhibition of emotional responses, likely by attenuating amygdala reactivity (39, 40).

Women with PMDD displayed decreased reactivity to negative social stimuli in the ACC, which are arguably more emotionally salient than isolated negative words (22). Furthermore, PMDD participants exhibited increased functional connectivity between the amygdala, insula, and ACC compared with controls, suggesting that bottom-up emotional reactivity predominates over top-down regulation in this population. Studies have also reported reduced pregenual ACC activation in women with PMDD during emotion processing tasks, independent of menstrual phase, indicating a trait-level alteration in emotion regulation networks (17).

In an empathy-induction study there was increased neural synchrony in the anterior cingulate cortex across PMDD patients compared with controls (23). An emotional discrimination study found increased activity of the left ACC in the luteal phase (28). Moreover, bilateral dorsal ACC activity was increased during passive viewing of aversive emotional stimuli independent of menstrual phase (29).

Together, this evidence points to impairments in top-down control mechanisms in PMDD, which may render affected individuals more vulnerable to emotionally charged stimuli and contribute to heightened emotional dysregulation.

#### 4.1.5 Amygdala

Among limbic structures, the amygdala has received the most extensive attention in PMDD neuroimaging because of its role in detecting and responding to emotionally salient stimuli, including socially significant information (41–44).

Compared with healthy controls and their own follicular phase, women with PMDD showed greater amygdala reactivity to social versus non-social stimuli in the luteal phase (22). Likewise, during the luteal phase, they exhibited increased amygdala responses to negative versus neutral stimuli in an emotional Go/No-Go task, indicating heightened premenstrual emotional reactivity (26). Significant changes in bilateral amygdala activity have also been observed during emotion-processing tasks, with increased activity in the follicular phase in PMDD subjects (20).

An important moderator of these patterns appears to be trait anxiety. Women with PMDD and high trait anxiety showed enhanced right amygdala responses in the luteal phase compared with controls and their own follicular phase, whereas women with PMDD and low trait anxiety showed higher right amygdala activity in the follicular phase (20). Healthy controls displayed higher left amygdala activation in the luteal than the follicular phase, a normal cyclic pattern absent in PMDD participants. This anxiety-dependent dissociation suggests that PMDD may comprise clinically meaningful subtypes, with implications for understanding heterogeneity in symptom presentation and treatment response.

It is important to note that some studies have not found consistent differences in amygdala activation between PMDD patients and controls (15, 24). These inconsistencies may stem from differences in stimulus type and task design. Taken together, these findings suggest that PMDD does not involve global amygdala dysfunction but rather dysregulation of specific corticolimbic circuits, particularly those engaged by socially relevant emotional information. This selectivity is consistent with clinical observations that socially salient situations frequently elicit disproportionate emotional responses in women with PMDD during the luteal phase, and amygdala hyperreactivity to social stimuli may represent a neural substrate underlying this symptom dimension.

#### 4.1.6 Cerebellum

Although the cerebellum has been traditionally associated with motor function, emerging evidence suggests a critical role for the cerebellum in emotion regulation, likely mediated through its connections with the amygdala, prefrontal cortex, and hypothalamus (45). It has recently gained attention in research on other psychiatric conditions including schizophrenia, bipolar disorder, depression, anxiety disorders, attention deficit hyperactivity disorder (ADHD), and autism (46). While evidence remains limited, recent studies implicate the role of the left posterior cerebellum in anger processing, whereas activation of the right posterior cerebellum is hypothesized to be associated with aggressive behavior (47–49).

Greater cerebellar activity was observed during emotional discrimination and emotion regulation tasks in the luteal phase and independent of the menstrual cycle phase, respectively, in women with PMDD compared with controls (28, 29). Hyperactivity of the left cerebellum during anger induction in women with PMDD may reflect exaggerated engagement of cerebellar circuits involved in processing negative emotions and threat detection, potentially contributing to heightened emotional sensitivity and dysregulation (19). Hypoactivity of the right cerebellum could reflect dysregulation of mechanisms involved in evaluation of aggressive behavior (50).

Further support for cerebellar involvement in PMDD comes from a PET study. Women with PMDD who were prospectively screened exhibited a larger increase in cerebellar activity, particularly in the midline vermis and fastigial nuclei, from the follicular phase to the symptomatic late luteal phase compared with healthy controls (51). The divergence between vermis hyperactivation in the PET study and the lateralized cerebellar findings in the anger-induction study may reflect functional specialization within cerebellar subregions (52).

Together, these findings suggest that cerebellar involvement in PMDD reflects affect-specific dysregulation across distinct subregions rather than a unitary disturbance, with potential relevance to the diverse emotional symptoms (irritability, anger, sadness) of the luteal phase. The cerebellum therefore warrants further investigation as a candidate substrate for the affective heterogeneity of PMDD.

#### 4.1.7 Nucleus accumbens

The nucleus accumbens (NAc) is a central component of the brain’s reward system, playing a pivotal role in processing pleasurable experiences and reinforcing behaviors associated with positive stimuli (53). PMDD subjects displayed a premenstrual decrease and a postmenstrual increase in nucleus accumbens response to positive words (26). This pattern is consistent with findings in major depression showing NAc hypoactivation to positive stimuli and may help explain reduced positive affect in women with PMDD (54).

#### 4.1.8 Insula

The insula is critical for integrating cognitive control, emotional salience, and interoceptive awareness with the anterior insula modulating activity in task-relevant brain regions as part of the salience network (55, 56). Beyond cognitive regulation, the insula is involved in the perception of subjective feelings of emotional state (57).

Compared with controls, women with PMDD in the luteal phase had enhanced insular reactivity to negative social versus non-social stimuli (22). In addition, PMDD subjects showed increased neural synchrony in the anterior insula during empathy induction and increased bilateral insula activity in an emotional discrimination task (23, 28). The insula integrates information about the significance of impending stimuli into perceptual decision-making during pain, and greater anterior insula activation is linked to higher predicted pain intensity (58, 59). This may explain insular hyperreactivity in PMDD, where increased activation could heighten perceived social distress and amplify emotional responses.

During a response inhibition task, PMDD patients showed reduced insula activation in the follicular phase and increased activation in the luteal phase, both relative to controls and to their own earlier-phase activity (16). This increased luteal-phase activation in PMDD could reflect compensatory over-recruitment of the insula in response to heightened emotional or interoceptive salience, whereas reduced follicular-phase activity may point to a baseline deficiency in cognitive-emotional control mechanisms.

Together, these patterns suggest that the anterior insula’s dynamic regulatory role is impaired in PMDD, contributing to the affective and cognitive symptoms that characterize the disorder. Given the insula’s role in translating interoceptive signals into subjective emotional experience, this dysregulation may contribute to the heightened sensitivity to interpersonal stressors, amplified physical symptom awareness, and emotional distress that characterize the luteal phase in PMDD. The involvement of the insula in the pathophysiology of PMDD is consistent with findings from studies of other psychiatric conditions, including mood disorders, panic disorder, post-traumatic stress disorder, obsessive–compulsive disorder, eating disorders, and schizophrenia (60).

### 4.2 Functional connectivity

The temporal cortex showed increased functional connectivity with the left executive control network before and after an emotion regulation task, with differences more robust after the task in PMDD compared with controls, independent of menstrual cycle phase; this may reflect a compensatory pathway for other malfunctioning networks (25). Moreover, the temporal cortex had increased connectivity with the striatum, thalamus, and pallidum (18). Conversely, the temporal cortex showed decreased functional connectivity with other cortical regions, including occipital, temporal, and prefrontal areas (26). Decreased connectivity of the temporal cortex, which is closely connected with limbic regions involved in emotional processing, with higher cortical regions may indicate poorer top-down regulation from prefrontal areas, reflecting higher emotional sensitivity in women with PMDD (61).

The amygdala showed increased connectivity with the right thalamus (25). Decreased connectivity was also found between the right amygdala and the left postcentral gyrus, and between the left amygdala and the bilateral precentral and postcentral gyri of the sensorimotor cortex. These connections are likely involved in pain processing (62). Given evidence that women with PMDD have higher sensitivity to pain, abnormalities in these connections may underlie the observed increased pain sensitivity (63).

### 4.3 Network analysis

#### 4.3.1 Salience network

The salience network, anchored by the anterior insula and anterior cingulate cortex (ACC), plays a pivotal role in detecting emotionally significant stimuli and directing attentional resources accordingly (64). The anterior insula serves as a key node for integrating cognitive control, emotional salience, and interoceptive awareness, while modulating activity in task-relevant brain regions (65). The ACC, particularly its ventral subdivision, contributes to emotional regulation through top-down inhibition of limbic responses, primarily by attenuating amygdala reactivity (40, 66). This coordinated network activity enables adaptive responses to emotionally salient information while maintaining regulatory control.

Evidence from multiple fMRI studies reveals marked disruptions in salience network function among women with PMDD, characterized by a pattern of heightened reactivity coupled with impaired regulatory control.

Women with PMDD demonstrate a complex pattern of ACC and insula dysfunctions that varies by task context and menstrual phase described in the previous section. Moreover, there was an increased functional connectivity between the ACC and insula, and amygdala and ACC in women with PMDD compared with healthy controls (22), suggesting a shift in the balance between bottom-up emotional reactivity and top-down regulatory control within this network.

Collectively, these findings point to a marked dysregulation of salience network functioning in PMDD characterized by exaggerated detection and responsivity to emotionally salient, particularly social, stimuli, compromised regulatory control from the ACC over limbic reactivity, and altered integration among network nodes, as evidenced by aberrant functional connectivity patterns. Rather than reflecting isolated regional hyper- or hypoactivation, PMDD appears to involve a systemic dysfunction in the salience network’s capacity to dynamically calibrate emotional salience with regulatory control across contexts.

#### 4.3.2 Executive control network

The executive control network (CEN), comprising the dorsolateral prefrontal cortex (dlPFC), posterior parietal cortex (PPC), frontal eye fields, and portions of the dorsomedial prefrontal cortex, mediates cognitive control, working memory, attentional allocation, and goal-directed behavior (67). Together, these regions form a lateralized network, typically right-hemisphere dominant for attention and bilateral for working memory that enables flexible cognitive control and the maintenance of goal-relevant information in the face of emotional distractors (68).

Research reveals substantial disruptions in executive control network function among women with PMDD, characterized by dlPFC dysfunction across multiple cognitive-emotional paradigms (15, 21, 24). Additionally, women with PMDD exhibited significantly stronger connectivity between a region of the left middle temporal gyrus and the left ECN relative to healthy controls when challenged by an emotional task (25).

While direct examination of posterior parietal cortex function in PMDD remains limited, the available evidence suggests potential alterations in attentional control systems. The PPC’s role in maintaining attention and filtering irrelevant information, particularly emotional distractors, may be compromised in PMDD (69). This could contribute to the heightened distractibility by emotional stimuli and difficulty maintaining focus on goal-relevant information that characterizes the disorder’s cognitive symptoms.

The executive control appears disrupted in PMDD, potentially explaining the cognitive difficulties, including concentration problems, indecisiveness, and mental fog, that many patients report during symptomatic periods. Further research specifically examining PPC and frontal eye field function during tasks requiring attentional control in the presence of emotional distractors would help clarify these networks’ contributions to PMDD symptomatology.

#### 4.3.3 Default mode network

Default mode network (DMN) comprised of the parahippocampal cortex, precuneus, posterior cingulate cortex, a caudal region of the inferior parietal lobule, the middle temporal cortex, the inferior fronto-lateral cortex, the superior frontal gyrus and anterior cingulate, as well as a small part of the middle frontal gyrus (70). The DMN is engaged when cognition shifts toward internally focused processes, including self-reflection and future-oriented simulation (71).

Research shows that women with PMDD exhibit hyperactivity of the posterior cingulate cortex, right superior frontal gyrus, and left precuneus during emotion discrimination task. Moreover, there was a decreased functional connectivity between posterior cingulate cortex and the middle temporal gyrus during emotional face-matching task as well as increased functional connectivity between the left executive control network and a region in the left middle temporal gyrus. Additionally, women with PMD, a milder form of PMDD, had decreased connectivity in the middle frontal gyrus and the parahippocampal gyrus and increased connectivity in the precentral gyrus and the left middle temporal gyrus/superior temporal (72).

This evidence points to dysfunctional connectivity both within the DMN and connectivity of DMN with other structures which might explain dysfunctional emotional regulation in women with PMDD and higher levels of self-focus of attention in response to stressors (73). Notably, women with PMDD use less helpful coping strategies such as rumination and increased self-focused attention in response to stress, suggesting that aberrant DMN connectivity may facilitate these maladaptive cognitive responses (74). Together, these patterns of findings suggest DMN involvement in PMDD’s pathophysiology.

#### 4.3.4 Network integration

Contemporary models of psychopathology increasingly emphasize that psychiatric symptoms emerge not from isolated regional dysfunction but from altered interactions among large-scale brain networks (75). The triple-network model proposes that adaptive cognitive-emotional functioning depends on coordinated activity among three core networks: the salience network (SN), which detects behaviorally relevant stimuli and initiates appropriate network responses; the executive control network (ECN), which supports goal-directed cognition and top-down regulation; and the default mode network (DMN), which enables self-referential processing and internally directed cognition (76).

In this framework, the salience network, particularly the anterior insula, serves as a dynamic switch that facilitates transitions between DMN-dominant states (during rest and self-reflection) and ECN-dominant states (during external task engagement) (77). Disruption of this switching mechanism has been implicated in multiple psychiatric conditions, including depression, anxiety disorders, and schizophrenia.

The neuroimaging findings reviewed above, when integrated within the triple-network framework, suggest that PMDD involves systematic disruption of intra- and inter-network dynamics rather than isolated dysfunction within any single brain area.

### 4.4 Mechanisms

The functional brain alterations documented in PMDD do not occur in isolation but rather emerge from, and interact with, well-characterized neurobiological processes. This section integrates the network-level findings presented above with established biological mechanisms implicated in PMDD pathophysiology, specifically: (1) altered sensitivity to progesterone-derived neurosteroids, particularly allopregnanolone; (2) GABAergic dysfunction; and (3) serotonergic dysregulation. Understanding these molecular-network interactions is essential for developing mechanistically informed therapeutic interventions.

#### 4.4.1 Allopregnanolone as a neuromodulator

Allopregnanolone (ALLO), a progesterone metabolite synthesized in both peripheral tissues and the brain, acts as a potent positive allosteric modulator of GABA-A receptors, enhancing chloride ion conductance and thereby exerting anxiolytic, sedative, and mood-stabilizing effects (78). ALLO levels rise substantially during the luteal phase, paralleling progesterone, and decline rapidly in the late luteal phase preceding menstruation.

Critically, PMDD is not characterized by abnormal hormone levels but rather by an aberrant CNS response to normal hormonal fluctuations (11). Women with PMDD appear to have altered sensitivity to ALLO, such that the neurosteroid’s normally calming effects are blunted or paradoxically reversed, contributing to the emergence of affective symptoms during the luteal phase (79).

Impaired ALLO-mediated GABAergic enhancement in the salience network could result in reduced inhibitory tone, leading to the heightened emotional salience detection and exaggerated reactivity to negative stimuli observed in PMDD. This mechanism aligns with findings that women with PMDD exhibit altered emotion-induced activation in salience network regions (insula, ACC) during the luteal phase, a period of maximal ALLO fluctuation and symptom severity.

The dorsolateral prefrontal cortex relies on GABAergic interneurons for precise temporal coordination of neural activity underlying working memory and cognitive control (80). Compromised ALLO-GABA-A signaling could disrupt this inhibitory scaffolding, impairing the dlPFC’s capacity to exert top-down regulation over limbic structures (81).

The amygdala is particularly enriched with GABA-A receptor subtypes sensitive to neurosteroid modulation, including those containing the δ subunit (78, 82). Altered ALLO sensitivity in PMDD could directly contribute to amygdala hyperreactivity by reducing tonic inhibition of amygdalar output neurons. The selective hyperreactivity to social-emotional stimuli during the luteal phase may reflect region-specific differences in GABA-A receptor composition and neurosteroid sensitivity within amygdala subnuclei (22).

Direct evidence linking neurosteroids to brain function in PMDD comes from combined hormonal-neuroimaging studies. Stiernman et al. (2023) examined emotion-induced brain activation across the menstrual cycle in women with PMDD and healthy controls, measuring serum levels of ALLO and its endogenous antagonist isoallopregnanolone (ISO) (28). Stiernman et al. (2023) found that emotion-induced activity in the right amygdala and parahippocampal gyrus correlated with the ISO/ALLO ratio in the late luteal phase, with a positive association in women with PMDD and a negative association in controls. This phase-specific divergence suggests that the balance between neurosteroid antagonist (ISO) and agonist (ALLO) activity modulates corticolimbic function differently in PMDD, potentially contributing to symptom expression during the symptomatic premenstrual period (28).

Furthermore, the therapeutic efficacy of sepranolone (isoallopregnanolone), a GABA-A receptor-modulating steroid antagonist that selectively blocks ALLO’s effects provides causal evidence that normalizing neurosteroid-GABA-A interactions can alleviate symptoms presumably by restoring adaptive network dynamics (83).

#### 4.4.2 GABAergic dysfunction and circuit imbalance

Proton magnetic resonance spectroscopy (1H-MRS) studies have directly quantified cortical GABA concentrations in women with PMDD, revealing alterations in GABAergic tone (84). Women with PMDD had markedly lower cortical GABA during the follicular phase compared with healthy controls, and while healthy women showed declining GABA levels from follicular to luteal phase, women with PMDD paradoxically showed increasing GABA levels across this interval. However, by the mid or late luteal phase GABA levels are not significantly different between these groups. Notably, the magnitude of cycle-related GABA change is significant in both groups, indicating that the group × phase interaction reflects opposite directional dynamics rather than a one-group phenomenon. These findings suggest a trait-level GABAergic abnormality that is most evident outside the symptomatic period (84), consistent with the broader proposal that PMDD pathophysiology is not restricted to the late luteal phase. A different study reported that women with PMDD had significantly lower GABA concentrations in both the anterior cingulate cortex/medial prefrontal cortex and the left basal ganglia during the late luteal phase, along with elevated glutamate-glutamine (Glx) levels in the ACC/mPFC (85). This pattern suggests not only reduced inhibitory tone but also a shift toward excitatory dominance in regions critical for emotional regulation. While these findings might appear contradictory, they may instead reflect complementary trait-level and state-level GABAergic features of PMDD: Epperson et al. captured a follicular-phase abnormality consistent with trait-level vulnerability, while Liu et al. captured a luteal-phase abnormality consistent with state-dependent dysregulation. Direct comparison is also limited by methodological differences, as Epperson et al. measured GABA in the occipital cortex at 2.1 T, while Liu et al. measured GABA in the ACC/mPFC and basal ganglia at 3 T.

The MRS findings map directly onto the network alterations documented in fMRI studies. The ACC, a central node of the salience network, shows both reduced GABA concentrations and altered functional activation in PMDD, both independently of cycle phase during emotion regulation and emotion processing tasks (17, 29), and specifically in the luteal phase during emotion discrimination, exposure to negatively-valenced stimuli, and empathy induction (22, 23, 28). Reduced GABAergic inhibition in the ACC would be expected to impair its capacity for top-down modulation of amygdala reactivity, consistent with the increased functional connectivity between ACC and amygdala observed in PMDD (22).

Beyond total GABA levels, the functional properties of GABAergic signaling depend critically on GABA-A receptor subunit composition. Receptors containing the δ subunit, which confer high sensitivity to neurosteroid modulation, show altered expression patterns in PMDD. Evidence suggests that women with PMDD have lower δ subunit mRNA expression during the luteal phase, and that this reduction correlates with increased amygdala activation (86).

This molecular finding provides a link between altered neurosteroid sensitivity and the amygdala hyperreactivity documented in fMRI studies. If δ-containing GABA-A receptors are downregulated, the inhibitory effects of rising ALLO during the luteal phase would be diminished, resulting in inadequate dampening of amygdala output and the emotional hyperreactivity characteristic of PMDD.

#### 4.4.3 Serotonergic mechanisms

Positron emission tomography (PET) studies using radioligands for serotonin receptors and transporters have revealed significant serotonergic alterations in PMDD. While healthy controls showed changes in 5-HT1A binding potential in the raphe nuclei from follicular to luteal phase, women with PMDD showed significantly smaller phase-related changes (87). This blunted serotonergic adaptation to menstrual cycle phase suggests impaired flexibility in the serotonin system’s response to hormonal fluctuations. Additionally, in a recent longitudinal study it was found that PMDD patients had significantly increased 5-HTT BPND, a marker of 5-HTT availability, in midbrain during the symptomatic premenstrual phase compared with the asymptomatic periovulatory phase as well as compared to controls (88). Moreover, there was a significant positive association between midbrain 5-HTT BPND levels and depressive symptom severity in patients across the menstrual cycle.

Serotonergic projections to the prefrontal cortex, particularly via 5-HT1A and 5-HT2A receptors on pyramidal neurons, modulate executive function and emotional regulation (89). The 5-HT1A receptor dysfunction documented in PMDD (87) could contribute to dlPFC hypofunction by impairing serotonergic facilitation of prefrontal inhibitory control. Notably, 5-HT2A receptors are densely expressed on prefrontal pyramidal neurons and their blockade enhances SSRI efficacy, suggesting that prefrontal serotonergic signaling is critical for emotional regulation (90).

The rapid efficacy of SSRIs in PMDD, often within one menstrual cycle, far faster than the 4–6 weeks required in major depression, suggests a mechanism distinct from traditional antidepressant effects. Emerging evidence indicates that SSRIs may exert their therapeutic effects in PMDD partly by enhancing neurosteroidogenesis, specifically by increasing the conversion of progesterone to ALLO (71, 91).

### 4.5 Limitations

Although overall methodological quality of included studies was good (13/15 scoring as low risk of bias on the Newcastle-Ottawa Scale), the most common weaknesses were inadequate reporting of non-response rates and limited descriptions of control selection procedures, which may have introduced selection bias. The relatively small sample sizes may have contributed to inconsistencies in findings across studies. The included fMRI studies were highly heterogeneous with respect to experimental paradigms and the definitions of menstrual cycle phases, which limits comparability across studies. Although resting-state fMRI is considered the most standardized approach, relatively few studies employed this methodology. Furthermore, some degree of overlap in study samples cannot be excluded, which may have biased the overall pattern of results. Studies examining PMDD in the context of comorbid psychiatric disorders were excluded, which reduced the number of eligible articles, as PMDD commonly co-occurs with other psychiatric conditions.

### 4.6 Conclusions

The pathophysiology of PMDD appears multifaceted and involves dysfunction across multiple brain regions. The present report suggests alterations in both top-down regulatory mechanisms and large-scale brain networks, including the salience network, default mode network, and executive control network. These alterations are characterized by decreased activation in the anterior cingulate cortex, dorsolateral prefrontal cortex, medial orbitofrontal cortex, and postcentral gyrus, alongside increased activation in the amygdala and insula, as well as impairments in corticolimbic connectivity. Such changes are likely driven by hypersensitivity to allopregnanolone (ALLO) and dysfunctions in GABAergic and serotonergic systems, including reduced δ subunit mRNA expression of GABA-A​ receptors. Notably, the cerebellum and insula, regions traditionally associated with motor control and autonomic function, respectively, also appear to play important roles in PMDD. Accumulating neuroimaging evidence indicates that these regions are similarly implicated in a range of other psychiatric disorders. The involvement of these regions therefore warrants further investigation and highlights promising directions for research into the neural abnormalities underlying neuropsychiatric conditions. In addition, further studies examining the functional organization of large-scale brain networks are needed to achieve a more comprehensive understanding of the pathophysiology of PMDD.

## Data Availability

The original contributions presented in the study are included in the article/**Supplementary Material**. Further inquiries can be directed to the corresponding author.
