# Supplementary material for: Functional MRI evidence of brain alterations in premenstrual dysphoric disorder: a systematic review
Source: Front Psychiatry. 2026 Jun 4;17:1795420. doi: 10.3389/fpsyt.2026.1795420 (PMC13275481; doi:10.3389/fpsyt.2026.1795420)
Supplement: Supplementary file 1 [file Table1.docx]

Table S1 PRISMA 2020 Checklist

| **Section and Topic** | **Item #** | **Checklist item** | **Location where item is reported** |
| --- | --- | --- | --- |
| **TITLE** | | | |
| **Title** | 1 | Identify the report as a systematic review. | Title page. The article title is "Functional MRI Evidence of Brain Alterations in Premenstrual Dysphoric Disorder: A Systematic Review." |
| **ABSTRACT** | | | |
| **Abstract** | 2 | See the PRISMA 2020 for Abstracts checklist. | Abstract (page 1). Reports background, methods, results, and conclusions. |
| **INTRODUCTION** | | | |
| **Rationale** | 3 | Describe the rationale for the review in the context of existing knowledge. | Section 1 (Introduction), paragraphs 1–4. Describes PMDD prevalence, broader menstrual health context, clinical impact, DSM-5/ICD-11 diagnostic criteria, PMS/PMDD distinction, treatment limitations (40% SSRI non-response), and rationale for fMRI as a relevant approach. |
| **Objectives** | 4 | Provide an explicit statement of the objective(s) or question(s) the review addresses. | Section 1 (Introduction), final paragraph. Aim stated: to summarize evidence on abnormal activity patterns and functional connectivity in women with PMDD as observed through fMRI, and to relate these neural alterations to the functions of affected brain regions. |
| **METHODS** | | | |
| **Eligibility criteria** | 5 | Specify the inclusion and exclusion criteria for the review and how studies were grouped for the syntheses. | Section 2.2 (Study selection criteria). Lists inclusion criteria (a–e) and exclusion criteria (a–e). Studies grouped narratively by brain region, functional connectivity, and large-scale network for synthesis. |
| **Information sources** | 6 | Specify all databases, registers, websites, organisations, reference lists and other sources searched or consulted to identify studies. Specify the date when each source was last searched or consulted. | Section 2.1 (Search strategy). PubMed, EMBASE, and Scopus searched; reference lists of included studies screened. Final search conducted in August 2025. |
| **Search strategy** | 7 | Present the full search strategies for all databases, registers and websites, including any filters and limits used. | Section 2.1 (Search strategy). Search terms: ("PMDD" OR "premenstrual dysphoric disorder") AND ("functional magnetic resonance" OR "fMRI"). English language limit applied (Section 2.2, inclusion criterion c). |
| **Selection process** | 8 | Specify the methods used to decide whether a study met the inclusion criteria of the review, including how many reviewers screened each record and each report retrieved, whether they worked independently, and if applicable, details of automation tools used in the process. | Section 2.4 (Data extraction). Two reviewers independently screened articles; disagreements resolved through discussion or third-party arbitration. Covidence software used for deduplication, screening, and data extraction (Section 2, Methods opening paragraph). |
| **Data collection process** | 9 | Specify the methods used to collect data from reports, including how many reviewers collected data from each report, whether they worked independently, any processes for obtaining or confirming data from study investigators, and if applicable, details of automation tools used in the process. | Section 2.4 (Data extraction). Two reviewers extracted data independently; disagreements resolved by discussion or third-party arbitration. Covidence used as the data collection platform. |
| **Data items** | 10a | List and define all outcomes for which data were sought. Specify whether all results that were compatible with each outcome domain in each study were sought (e.g. for all measures, time points, analyses), and if not, the methods used to decide which results to collect. | Section 2.4 (Data extraction). Outcomes sought: evidence of neuroimaging alterations (BOLD activation, functional connectivity, regional homogeneity, neural synchrony) across all reported brain regions, tasks, and menstrual cycle phases. |
|  | 10b | List and define all other variables for which data were sought (e.g. participant and intervention characteristics, funding sources). Describe any assumptions made about any missing or unclear information. | Section 2.4 (Data extraction). Variables extracted: author, year, sample size, control group presence, group sizes, mean age, PMDD diagnostic criteria, menstrual cycle phases at scanning, methods to confirm cycle stage, image analysis techniques, regions investigated, and task performance. |
| **Study risk of bias assessment** | 11 | Specify the methods used to assess risk of bias in the included studies, including details of the tool(s) used, how many reviewers assessed each study and whether they worked independently, and if applicable, details of automation tools used in the process. | Section 2.3 (Risk of Bias assessment). Newcastle-Ottawa Scale (NOS) for case-control studies used; for the comparability domain, absence of current hormonal contraceptive use and absence of current Axis-I psychiatric diagnoses were pre-specified as matching factors; "exposure" domain operationalized as PMDD diagnosis with prospective daily symptom ratings as gold-standard ascertainment. Two reviewers assessed each study independently; disagreements resolved by discussion. Studies scoring 7–9 stars classified as low risk, 4–6 moderate, 0–3 high. Full assessment in Supplementary Table S1. |
| **Effect measures** | 12 | Specify for each outcome the effect measure(s) (e.g. risk ratio, mean difference) used in the synthesis or presentation of results. | Not applicable. No meta-analysis was performed; narrative synthesis of qualitative imaging findings reported as direction of activation difference (↑/↓ BOLD, FC, ReHo, NS) between PMDD and controls and between cycle phases (Table 1). |
| **Synthesis methods** | 13a | Describe the processes used to decide which studies were eligible for each synthesis (e.g. tabulating the study intervention characteristics and comparing against the planned groups for each synthesis (item #5)). | Section 2.2 + 2.4 + Section 3.2. All eligible studies meeting PICO inclusion criteria were included in narrative synthesis; studies were grouped by analysis type (region-specific, functional connectivity, large-scale network) for the Discussion. |
|  | 13b | Describe any methods required to prepare the data for presentation or synthesis, such as handling of missing summary statistics, or data conversions. | Not applicable. No quantitative data conversion was required for narrative synthesis. Findings extracted as reported by primary studies. |
|  | 13c | Describe any methods used to tabulate or visually display results of individual studies and syntheses. | Section 3 (Results), Table 1 presents study characteristics and main findings for all 15 included studies, including sample size, neuroimaging technique, task, between-group results, and within-group cycle-phase results. |
|  | 13d | Describe any methods used to synthesize results and provide a rationale for the choice(s). If meta-analysis was performed, describe the model(s), method(s) to identify the presence and extent of statistical heterogeneity, and software package(s) used. | Section 4 (Discussion) and Section 4.6 (Limitations). Narrative synthesis was used due to substantial heterogeneity in fMRI paradigms, analysis pipelines, and definitions of menstrual cycle phases across studies, which precluded meta-analysis. |
|  | 13e | Describe any methods used to explore possible causes of heterogeneity among study results (e.g. subgroup analysis, meta-regression). | Not applicable to formal statistical exploration. Sources of heterogeneity (paradigm differences, cycle-phase definitions, sample overlap) discussed narratively in Section 4.6 (Limitations). |
|  | 13f | Describe any sensitivity analyses conducted to assess robustness of the synthesized results. | Not applicable. No quantitative sensitivity analyses performed given narrative synthesis. |
| **Reporting bias assessment** | 14 | Describe any methods used to assess risk of bias due to missing results in a synthesis (arising from reporting biases). | Not formally assessed. No funnel plot or statistical test of publication bias performed, as no meta-analysis was conducted. The potential for publication bias is acknowledged in Section 4.6 (Limitations). |
| **Certainty assessment** | 15 | Describe any methods used to assess certainty (or confidence) in the body of evidence for an outcome. | Not formally assessed using GRADE or comparable framework. Strength of evidence discussed narratively in Section 4 (Discussion) and limitations addressed in Section 4.6. |
| **RESULTS** | | | |
| **Study selection** | 16a | Describe the results of the search and selection process, from the number of records identified in the search to the number of studies included in the review, ideally using a flow diagram. | Section 3.1 (Literature search). 521 records identified (PubMed: 22; EMBASE: 62; Scopus: 437) plus 1 record from citation searching; 69 duplicates removed; 437 excluded at title/abstract screening; 16 sought for retrieval, 1 not retrieved; 15 included. PRISMA flow diagram presented as Figure 1 in the main manuscript. |
|  | 16b | Cite studies that might appear to meet the inclusion criteria, but which were excluded, and explain why they were excluded. | Section 3.1 (Literature search). 437 records excluded at title/abstract screening as irrelevant; 1 record not retrieved. No further studies excluded at full-text eligibility stage. |
| **Study characteristics** | 17 | Cite each included study and present its characteristics. | Section 3.2 (Characteristics of the included studies) and Table 1. All 15 included studies are cited with sample sizes, neuroimaging technique, task, and main findings. |
| **Risk of bias in studies** | 18 | Present assessments of risk of bias for each included study. | Section 3.2 (Characteristics of the included studies) summarises results: 13 of 15 studies scored 7–9 NOS stars (low risk of bias); 2 studies scored 6 stars (moderate risk); all studies met case definition and comparability criteria; most consistent weaknesses were inadequate reporting of non-response rates and limited descriptions of control selection procedures. Full per-study assessment provided in Supplementary Table S1. |
| **Results of individual studies** | 19 | For all outcomes, present, for each study: (a) summary statistics for each group (where appropriate) and (b) an effect estimate and its precision (e.g. confidence/credible interval), ideally using structured tables or plots. | Section 3 (Results), Table 1. Direction of effect (↑/↓ activation, FC, ReHo, NS) for each included study reported by region and task; primary studies did not consistently report effect sizes amenable to summary, so qualitative direction is presented. |
| **Results of syntheses** | 20a | For each synthesis, briefly summarise the characteristics and risk of bias among contributing studies. | Section 3.2 (study characteristics and NOS summary) and Section 3.3 (Main findings). Overall methodological quality was good (13/15 low risk of bias); main findings synthesised across all included studies. |
|  | 20b | Present results of all statistical syntheses conducted. If meta-analysis was done, present for each the summary estimate and its precision (e.g. confidence/credible interval) and measures of statistical heterogeneity. If comparing groups, describe the direction of the effect. | Not applicable. No meta-analysis performed. Direction of effect for each study reported in Table 1 and described narratively in Section 3.3 (Main findings) and Section 4 (Discussion). |
|  | 20c | Present results of all investigations of possible causes of heterogeneity among study results. | Not applicable. Sources of heterogeneity discussed narratively in Section 4.6 (Limitations). |
|  | 20d | Present results of all sensitivity analyses conducted to assess the robustness of the synthesized results. | Not applicable. |
| **Reporting biases** | 21 | Present assessments of risk of bias due to missing results (arising from reporting biases) for each synthesis assessed. | Not formally assessed (see item 14). Possibility of publication bias acknowledged in Section 4.6 (Limitations). |
| **Certainty of evidence** | 22 | Present assessments of certainty (or confidence) in the body of evidence for each outcome assessed. | Not formally assessed (see item 15). Strength of evidence discussed narratively throughout Section 4 (Discussion). |
| **DISCUSSION** | | | |
| **Discussion** | 23a | Provide a general interpretation of the results in the context of other evidence. | Section 4 (Discussion), subsections 4.1–4.4. Findings interpreted by brain region, functional connectivity, large-scale networks (triple-network model), and integrated with biochemical mechanisms (allopregnanolone, GABAergic, serotonergic). |
|  | 23b | Discuss any limitations of the evidence included in the review. | Section 4.6 (Limitations). Heterogeneity of fMRI paradigms and cycle-phase definitions, small sample sizes, sample overlap across studies, limited use of resting-state methodology, and exclusion of comorbid PMDD studies discussed. |
|  | 23c | Discuss any limitations of the review processes used. | Section 4.6 (Limitations). English-language limit and absence of grey literature search acknowledged as review-process limitations. |
|  | 23d | Discuss implications of the results for practice, policy, and future research. | Section 4.7 (Conclusions) and throughout Section 4 (Discussion). Implications include identification of large-scale network dysfunction as therapeutic target, mechanistic relevance for novel interventions (e.g., sepranolone), and need for further research on cerebellum, insula, and large-scale network organization in PMDD. |
| **OTHER INFORMATION** | | | |
| **Registration and protocol** | 24a | Provide registration information for the review, including register name and registration number, or state that the review was not registered. | Title page, Abstract, and Section 2 (Methods). Registered prospectively in PROSPERO; registration number CRD420251174749. |
|  | 24b | Indicate where the review protocol can be accessed, or state that a protocol was not prepared. | Protocol accessible via PROSPERO database (CRD420251174749) at https://www.crd.york.ac.uk/prospero/. |
|  | 24c | Describe and explain any amendments to information provided at registration or in the protocol. | No substantive amendments to the registered protocol. |
| **Support** | 25 | Describe sources of financial or non-financial support for the review, and the role of the funders or sponsors in the review. | Funding statement (front matter) and Acknowledgments section. The authors received no external funding or financial support for this work. |
| **Competing interests** | 26 | Declare any competing interests of review authors. | Conflict of Interest section (front matter and end of manuscript). The authors declare no commercial or financial relationships that could be construed as a potential conflict of interest. |
| **Availability of data, code and other materials** | 27 | Report which of the following are publicly available and where they can be found: template data collection forms; data extracted from included studies; data used for all analyses; analytic code; any other materials used in the review. | Data Availability Statement (front matter). The original contributions are included in the article and supplementary material (Supplementary Table S1: full NOS assessment; Supplementary File S2: PRISMA 2020 checklist); further inquiries can be directed to the corresponding author. |

*Page MJ, McKenzie JE, Bossuyt PM, Boutron I, Hoffmann TC, Mulrow CD, et al. The PRISMA 2020 statement: an updated guideline for reporting systematic reviews. BMJ 2021;372:n71. doi: 10.1136/bmj.n71. Licensed under CC BY 4.0.*
