# Supplementary material for: Functional MRI evidence of brain alterations in premenstrual dysphoric disorder: a systematic review
Source: Front Psychiatry. 2026 Jun 4;17:1795420. doi: 10.3389/fpsyt.2026.1795420 (PMC13275481; doi:10.3389/fpsyt.2026.1795420)
Supplement: Supplementary file 2 [file Table2.docx]

Table S2 Risk of bias assessment of included studies using the Newcastle-Ottawa Scale (NOS) for case-control studies.

| **Study** | **Selection (max 4** ★**)** | | | | **Comparability (max 2** ★**)** | **Exposure (max 3** ★**)** | | | **Total** | **Risk of bias** |
| --- | --- | --- | --- | --- | --- | --- | --- | --- | --- | --- |
|  | **S1** | **S2** | **S3** | **S4** |  | **E1** | **E2** | **E3** |  |  |
| Baller et al., 2013 | ★ | ★ | ★ | ★ | ★★ | ★ | ★ | — | **8/9** | Low |
| Bannbers et al., 2012 | ★ | ★ | — | ★ | ★★ | ★ | ★ | — | **7/9** | Low |
| Comasco et al., 2014 | ★ | ★ | — | ★ | ★★ | ★ | ★ | — | **7/9** | Low |
| Dan et al., 2020 | ★ | ★ | ★ | ★ | ★★ | ★ | ★ | ★ | **9/9** | Low |
| Gao et al., 2021 | ★ | ★ | ★ | ★ | ★★ | — | — | — | **6/9** | Moderate |
| Gingnell et al., 2012 | ★ | ★ | — | ★ | ★★ | ★ | ★ | — | **7/9** | Low |
| Gingnell et al., 2013 | ★ | ★ | — | ★ | ★★ | ★ | ★ | — | **7/9** | Low |
| Gingnell et al., 2014 | ★ | ★ | — | ★ | ★★ | ★ | ★ | — | **7/9** | Low |
| Lerner et al., 2024 | ★ | ★ | — | ★ | ★★ | ★ | ★ | — | **7/9** | Low |
| Petersen et al., 2018 | ★ | ★ | ★ | ★ | ★★ | ★ | ★ | — | **8/9** | Low |
| Petersen et al., 2019 | ★ | ★ | ★ | ★ | ★★ | ★ | ★ | — | **8/9** | Low |
| Protopopescu et al., 2008 | ★ | — | — | ★ | ★★ | ★ | ★ | — | **6/9** | Moderate |
| Reuveni et al., 2023 | ★ | ★ | ★ | ★ | ★★ | ★ | ★ | ★ | **9/9** | Low |
| Stiernman et al., 2023 | ★ | ★ | ★ | ★ | ★★ | ★ | ★ | — | **8/9** | Low |
| Stiernman et al., 2025 | ★ | ★ | ★ | ★ | ★★ | ★ | ★ | — | **8/9** | Low |

**Notes.** Each domain item was scored as awarded (★) or not awarded (—). **Selection:** S1, case definition adequate; S2, representativeness of cases; S3, selection of controls; S4, definition of controls. **Comparability:** two stars awarded when studies controlled for absence of current hormonal contraceptive use and absence of current Axis-I psychiatric diagnoses. **Exposure:** E1, ascertainment of exposure; E2, same method of ascertainment for cases and controls; E3, non-response rate. **Reference:** Wells GA, Shea B, O’Connell D, Peterson J, Welch V, Losos M, Tugwell P. The Newcastle-Ottawa Scale (NOS) for assessing the quality of nonrandomised studies in meta-analyses. Ottawa: Ottawa Hospital Research Institute. <https://ohri.ca/en/who-we-are/core-facilities-and-platforms/ottawa-methods-centre/newcastle-ottawa-scale> Accessed in May 2026
